# Serum 25-hydroxyvitamin D level and erectile dysfunction: a causal relationship? Findings from a two-sample Mendelian randomization study

**DOI:** 10.3389/fmolb.2024.1390814

**Published:** 2024-06-12

**Authors:** Hengchang Sun, Gang Shen, Huimin Dong, Mei Shang, Wenying Zhou, Lingling Wang, Zhaoxia Li, Jiao Gong, Bo Hu

**Affiliations:** Department of Laboratory Medicine, Third Affiliated Hospital of Sun Yat-sen University, Guangzhou, China

**Keywords:** serum 25-hydroxyvitamin D, erectile dysfunction, Mendelian randomization, causal effect, genetic variant

## Abstract

**Background:**

Serum 25-hydroxyvitamin D level is associated with erectile dysfunction (ED) in observational studies. However, whether there is a causal association between them remains uncertain.

**Objective:**

Conduct a two-sample Mendelian randomization (MR) analysis to investigate the causal effect between serum 25-hydroxyvitamin D level and ED risk.

**Method:**

Genome-wide association study (GWAS) data of serum 25-hydroxyvitamin D levels comprising 6,896,093 single nucleotide polymorphisms (SNP) from 496,949 people of European ancestry were regarded as exposure for the MR analysis. Additional GWAS data involving 9,310,196 SNPs of 6,175 European ED cases and 217,630 controls were used as outcome data. The MR-Egger, inverse variance weighted (IVW) method, weighted median, simple mode, and weighted mode were employed to evaluate causal effects, among which IVW was the primary MR analysis method. The stability of the MR analysis results was confirmed by a heterogeneity test, a horizontal pleiotropy test, and the leave-one-out method.

**Result:**

There were 103 SNPs utilized as instrumental variables (*p* < 5 × 10^−8^). The results of MR analysis showed no causal effects of serum 25(OH) D concentration on ED risks (IVW; OR = 0.9516, 95% CI = 0.7994 to 1.1328, *p* = 0.5772). There was no heterogeneity and pleiotropy in the statistical models.

**Conclusion:**

The present MR study did not support a causal association for genetically predicted serum 25-hydroxyvitamin D concentration in the risk of ED in individuals of European descent.

## Introduction

Erectile dysfunction (ED) is characterized by the inability to achieve or maintain an erection sufficient for satisfactory sexual performance (Shamloul and Ghanem, 2013). As reported in the Massachusetts Male Aging Study (MMAS), ED was reported in over 52% of men aged between 40 and 70, and ED morbidity reached about 26 cases per 1,000 man-years (Johannes et al., 2000). The prevalence of ED increased with age; the morbidity was approximately 6% in men under 49 years old and 16% in men aged 50–59. This prevalence rate increased to 32% for men aged 60–69 and 44% for men aged 70–79 (Eardley, 2013). The number of ED cases was estimated to reach 322 million worldwide by 2025 (Ayta et al., 1999). ED has a profound impact on the quality of life, self-esteem, and intimate relationships of those affected (Shamloul and Ghanem, 2013). Hence, early intervention and prevention of ED is urgently needed and significant. As researchers continue to investigate potential risk factors and underlying mechanisms associated with ED, the role of vitamin D has emerged as an area of interest.

Vitamin D is primarily obtained through exposure to sunlight and can also be acquired through dietary sources or supplementation (Canguven and Al Malki, 2021). Accordingly, vitamin D deficiency has been a pandemic health problem in developing and developed countries (Palacios and Gonzalez, 2014). Increasing numbers of studies showed that vitamin D has a causal association with various diseases (Zhou et al., 2022; Zhao and Burgess, 2023; Zhao et al., 2023; Zhou and Hyppönen, 2023). It is converted into its active form, calcitriol, which binds to vitamin D receptors in various tissues, including those involved in erectile function. Several studies have explored the potential link between vitamin D deficiency and the risk of ED (Barassi et al., 2014; Basat et al., 2018; Arıman et al., 2021; Canguven and Al Malki, 2021; Kim and Cho, 2022). Some have suggested that low levels of vitamin D may contribute to endothelial dysfunction, inflammation, and impaired nitric oxide production, all of which are factors that contribute to the development of ED (Crafa et al., 2023). Barassi et al. (2014) found that ED patients with an arteriogenic etiology have vitamin D deficiency more frequently. Inversely, a meta-analysis (Wei et al., 2019) disclaimed the relationship between vitamin D and the risk of ED. Another study did not support the association of serum 25-hydroxyvitamin D level concentration with the presence and severity of ED in renal transplant recipients (Sudarević et al., 2017). Hence, the evidence regarding the direct causal relationship between vitamin D and ED remains inconclusive.

Given this uncertainty, we used a new approach to address the bias from observational design, employing two-sample Mendelian randomization (MR) to investigate the causal association of serum 25-hydroxyvitamin D level with ED. MR typically utilizes genetic variation (usually single nucleotide polymorphisms, or SNPs) to assess the causal relationship between exposure (e.g., sugar intake) and an outcome (e.g., obesity). Because SNPs are randomly allocated among individuals, they are not affected by environmental or lifestyle factors and also do not change over a lifetime. Using SNPs as a proxy can reduce bias due to confounding and reverse causation, which is commonly seen in observational studies (Davies et al., 2018). Hence, MR is considered a natural analog of the classical randomized controlled trial (RCT) (Davies et al., 2018). It has been increasingly used to investigate the causal evidence for different exposures (Xi et al., 2023; Xiong et al., 2023; Ye et al., 2023), where it is either unethical or infeasible to conduct RCTs.

Recently, the explosive growth of genome-wide association studies revealed that serum 25 hydroxyvitamin D levels and some diseases, such as possibly ED, are closely associated with several gene variants (SNPs) (Bovijn et al., 2019; Revez et al., 2020). An improved understanding of the genetic determinants of 25OHD has helped reassess the role of vitamin D in the etiology of various diseases, such as musculoskeletal disorders, autoimmune diseases, and cancer (Manousaki et al., 2020). Moreover, various MR studies were conducted for causal effect estimation by utilizing genetic variants associated with 25OHD (Zhou et al., 2022; Zhang X. et al., 2023; Zhao and Burgess, 2023; Zhao et al., 2023; Zhou and Hyppönen, 2023).

In this study, we employed a two-sample MR approach to investigate the causal association between serum 25-hydroxyvitamin D (25-hydroxyvitamin D, an established marker for nutritional vitamin D status) levels and ED risk. To the best of our knowledge, this is the first study exploring the causal relationship between serum 25-hydroxyvitamin D level and the ED risk by MR analysis.

## Method and material

### Study design

**FIGURE 1:**
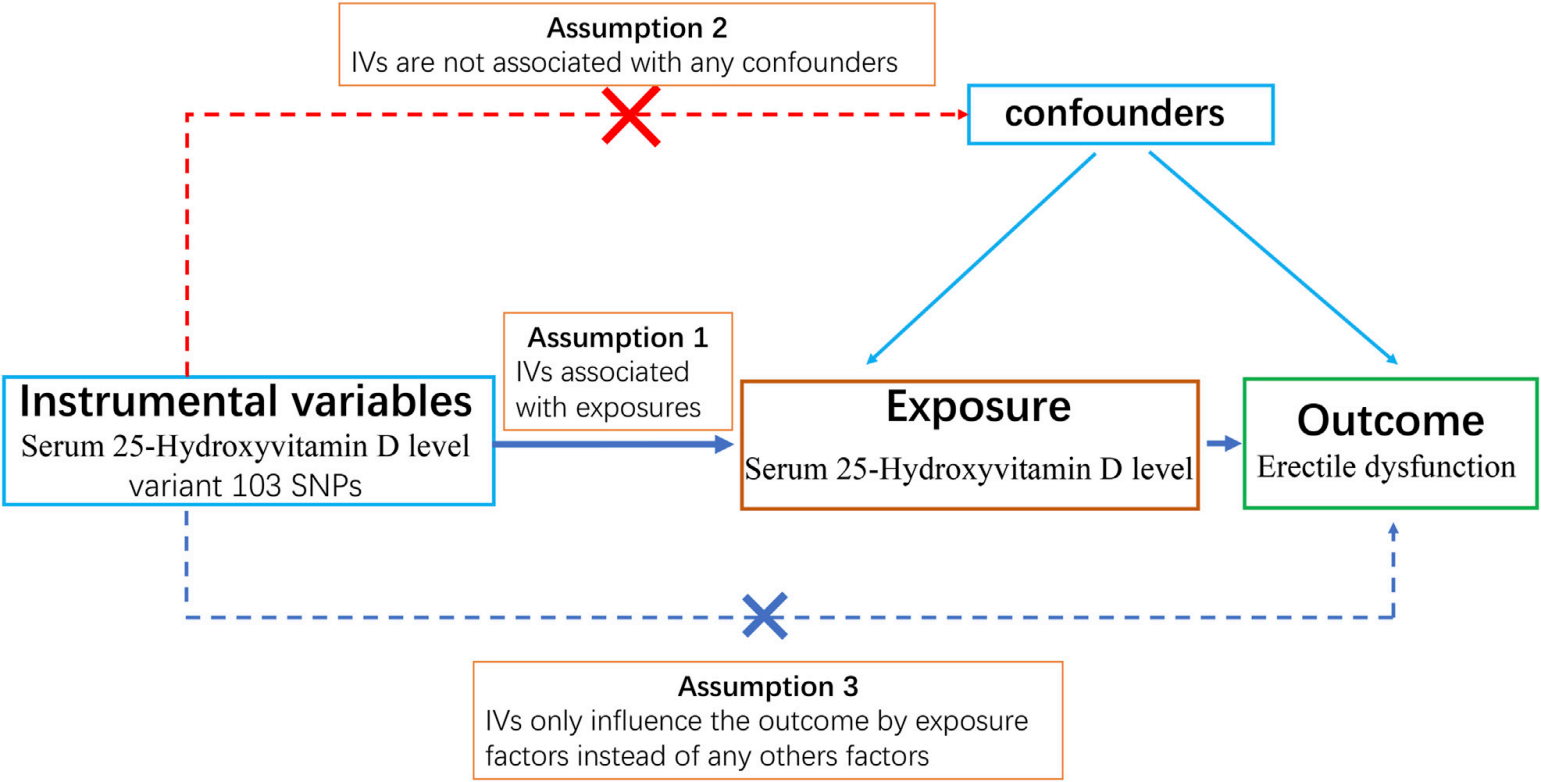
Diagram of the three key assumptions and design of the Mendelian randomization study.

We designed a two-sample MR study to obtain a comprehensive and reliable conclusion of the causal link between serum 25-hydroxyvitamin D levels (exposure) and ED (outcome). A schematic summary of the study design is given in Figure 1.

### GWAS data of serum 25-hydroxyvitamin D levels and erectile dysfunction

The exposure genome-wide association study (GWAS) data of serum 25-hydroxyvitamin D levels (496,946 European samples, 6,896,093 SNPs) were obtained from a publicly available database (IEU OpenGWAS Project, https://gwas.mrcieu.ac.uk, ID: ebi-a-GCST90000618). The subjects of this GWAS data were of European ancestry (Revez et al., 2020).

GWAS summary data for ED were downloaded from the IEU OpenGWAS Project database (GWAS ID: ebi-a-GCST006956), which contains 9,310,196 SNPs of 6,175 ED cases and 217,630 control samples (Bovijn et al., 2019). The participants in this summary data were also of European ancestry. They were from three cohorts: the United Kingdom Biobank (UKB), the Estonian Genome Center of the University of Tartu (EGCUT) cohort, and the hospital-recruited Partners Healthcare Biobank (PHB) cohort. ED was diagnosed according to codes of International Classification of Diseases version 10 (N48.4 and F52.2), oral medication history (e.g., sildenafil), surgical intervention, or respondents’ self-report. The prevalence of ED was 1.5%, 7.0%, and 25.4% in the UKB, EGCUT, and PHB cohorts, respectively (Bovijn et al., 2019). Ethical approval was waived for this research, and all subjects in the original GWAS have provided informed consent.

### Selection of the genetic instrument

Three core assumptions must be satisfied to obtain reliable results for a Mendelian randomization study: 1) The genetic instrument must be strongly associated with exposure factors (i.e., 25-OH-VD) (relevance assumption). Typically, the genetic variants selected as instrumental variables must be strongly associated with risk factors at traditional genome-wide significance levels (*p* < 5 × 10^−8^), or the genetic variants have been proven to be closely associated with exposure factors by gene function experiments; 2) The selected genetic instrument must not be associated with confounders of the association between exposures and outcome (independence assumption); 3) Genetic variation only influences the outcome by exposure factors (exclusivity assumption) (Lawlor et al., 2008). For example, we want to study if sugar intake (exposure) leads to obesity (outcome) by MR analysis. The SNPs we select as genetic instruments must be able to increase the sugar intake of individuals. Additionally, these SNPs must not be associated with confounding factors such as unhealthy habits (e.g., exercising less or going to bed later) that can cause obesity. Moreover, these SNPs must only influence obesity by increasing sugar intake instead of directly influencing obesity; otherwise, we cannot be sure if sugar consumption causes obesity or if SNPs directly cause obesity.

Based on the above rules, SNPs with a strong association with VD level were filtered based on the following criteria: genome-wide significance *p* < 5 × 10^−8^ and linkage disequilibrium *r* ^*2*^ < 0.001 within a 10,000 kb window. In addition, to avoid bias from weak IVs, the F-statistics of SNPs were calculated using the following formula: F-statistics = (Beta/Se)^2^ (Li et al., 2023). The values of F-statistics represented the strength of IVs, and generally, F-statistics <10 were deleted as weak IVs. The filtered SNPs were finally qualified for the following MR analysis.

### Elimination of confounding factors

We examined PhenoScanner (www.phenoscanner.medschl.cam.ac.uk) for potential confounders, including prostate cancer, diabetes, depression, and bipolar disorder. SNPs associated with any of these potential confounders on a genome-wide basis were dropped in the following MR analysis.

### Statistical analysis

In this MR study, all the analyses were performed by the R software (version 4.2.0, http://www.R-project.org; The R Foundation, Vienna, Austria), with the “TwoSampleMR” packages (version 0.5.6). Five different methods were employed: the inverse variance weighted (IVW), MR-Egger, weighted median, weighted mode, and simple mode (Zhang K. et al., 2023). The IVW method assumes that all the SNPs in the MR analysis are valid and then combines the Wald ratio of each SNP into an overall weighted effect. Results generated by the IVW method were regarded as the main findings (Guo et al., 2022).

Mendelian randomization-Egger (MR-Egger) is an analysis method for MR using summarized genetic data. The MR-Egger method enables us to assess whether genetic variants have pleiotropic effects on the outcome that differ on average from zero (directional pleiotropy). We utilized the MR-PRESSO global test and MR-Egger regression to evaluate the pleiotropy of IVs, and *p* < 0.05 represents having pleiotropy. The heterogeneity between the genetic instruments was evaluated by applying Cochran’s Q test. The leave-one-out analysis was also utilized to analyze whether influential SNPs existed in causal links between VD level and ED (Burgess et al., 2017).

## Results

### SNPs used as instrumental variables

**FIGURE 2:**
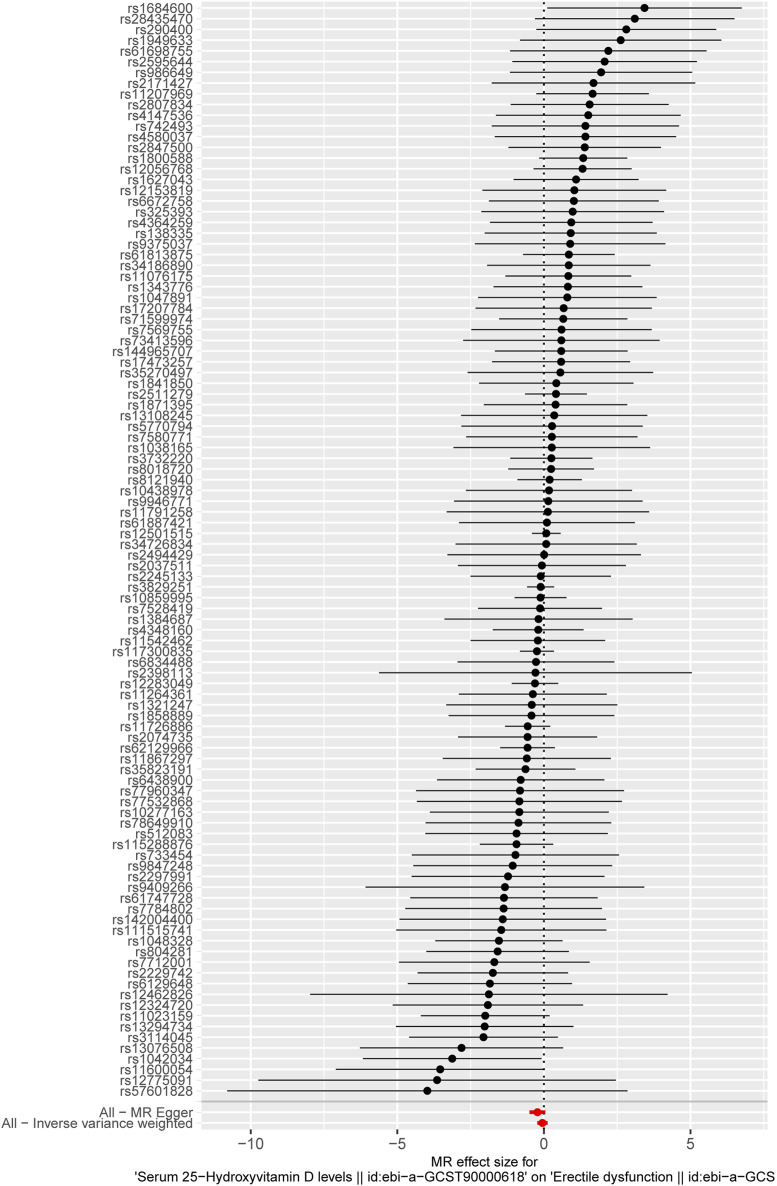
The forest plot displays the causal impact of each SNP on ED. The X-axis represents the effect of each SPN on ED, and the Y-axis represents the SNPs. Each horizontal solid line reflects a single SNP. The solid black lines located on the left side of 0 indicate that the result estimated by this SNP is that increased VD reduces the risk of ED, and solid lines on the right side of 0 indicate that the result estimated by this SNP is that the increase in VD increases the risk of ED. Meanwhile, the lines that cross over 0 represent effects that are not obvious. The bottom red lines represent the combined analysis results of all the SNPs.

Based on the criteria of the instrumental SNP selection, 117 LD-independent SNPs were obtained from the 25-hydroxyvitamin D GWAS. Fourteen SNPs associated with possible confounders of ED, such as prostate cancer (1 SNP), diabetes (11 SNPs), depression, and bipolar disorder (2 SNPs), were excluded in the following MR analysis (Supplementary Table S1). The remaining 103 SNPs could be extracted from the GWAS of ED. The F-statistics of these 103 SNPs were >10, showing a low likelihood of weak IV bias (Supplementary Table S2). The forest plot shows a causal impact on the ED of each SNP (Figure 2). In Figure 2, the *X*-axis represents the effect of each SNP on ED, and the *Y*-axis represents SNPs. Each horizontal solid line reflects a single SNP. A horizontal solid black line located completely on the left side of 0 indicates that the result estimated by this SNP is that increased VD reduces the risk of ED, and a solid line completely on the right side of 0 indicates that the result estimated by this SNP is that the increase of VD increases the risk of ED. Meanwhile, the lines that cross over 0 represent effects that are not obvious. However, the results of a single SNP are not robust because the serum VD level here is affected by multiple SNPs, so it is necessary to combine the results; that is the bottom red line. As indicated by the forest plot, the combined analysis results (the bottom red line) cross over 0, representing serum 25-hydroxyvitamin D level has no causal effect on ED.

### Mendelian randomization test results

**FIGURE 3:**
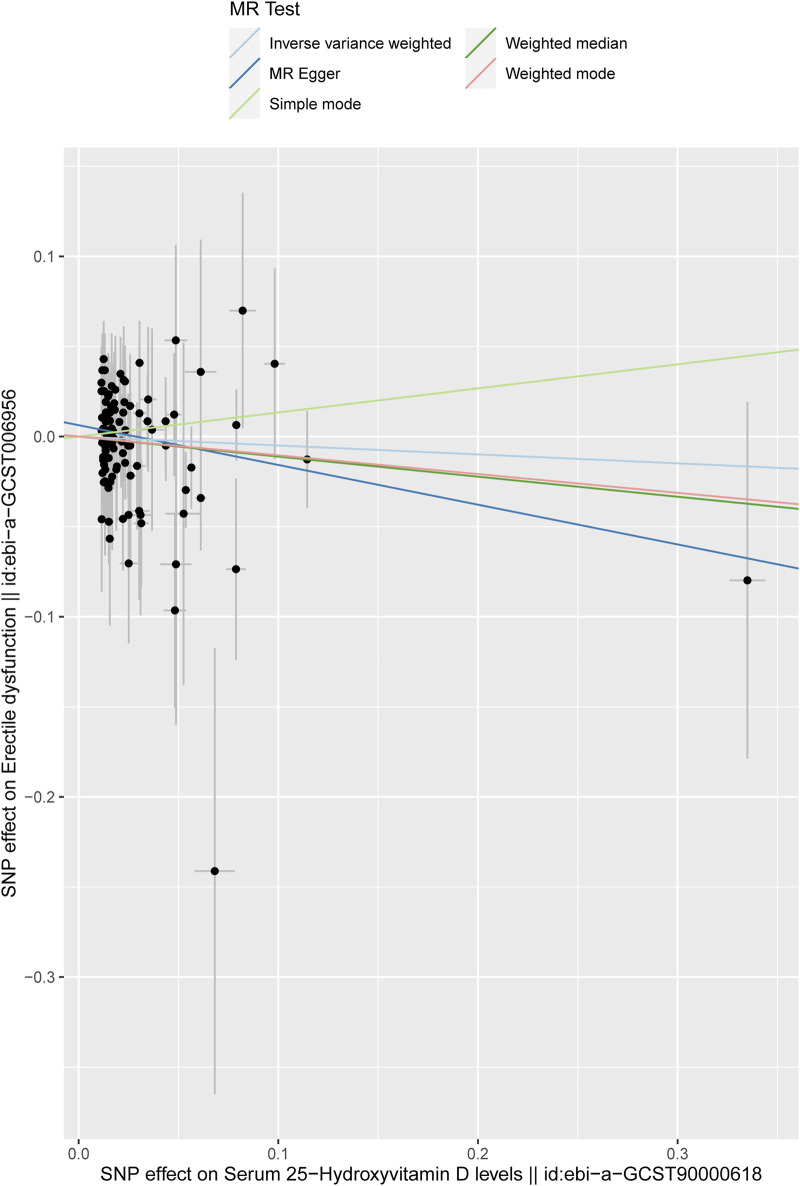
Scatter plot of the effect size for each SNP on serum 25-hydroxyvitamin D levels and ED. The *X*-axis represents the effects of SNPs on serum 25-hydroxyvitamin D, and the *Y*-axis represents the effects of SNPs on ED. Each point represents one SNP. The ratio of the two effects is the effect of serum 25-hydroxyvitamin D on ED, which is the slope of the colored lines. The different colored lines represent different MR algorithms.

In this study, the result of the IVW method was deemed the main finding for the causal effects. It was found that no causal effects were associated between the serum 25-hydroxyvitamin D level and ED (IVW; odds ratio (OR):0.9516, 95% CI: 0.7994–1.1328, *p* = 0.5772). Then, the other four different models were adopted to test and verify the causal relationship between serum 25-hydroxyvitamin D level and ED. The results of MR-Egger regression, weighted median, simple model, and weighted model were consistent with the IVW result (Table 1), as shown in the scatter plot (Figure 3). In Figure 3, the *X*-axis represents the effects of SNPs on serum 25-hydroxyvitamin D, and the *Y*-axis represents the effects of SNPs on ED. Each point represents one SNP. The ratio of the two effects is the effect of serum 25-hydroxyvitamin D on ED, which is the slope of the colored lines. The lines of different colors represent different MR algorithms. The results of the five different MR algorithms did not supports the causal effect of serum 25-hydroxyvitamin D level on ED risk (*p* > 0.05).

**TABLE 1:** Associations between the serum 25-hydroxyvitamin D level and risk of erectile dysfunction.

Method	Or (95%CI)	*p*-value
MR-Egger	0.802 (0.612–1.051)	0.113
Weighted median	0.895 (0.683–1.172)	0.419
Inverse variance weighted	0.952 (0.799–1.133)	0.577
Simple mode	1.143 (0.638–2.047)	0.654
Weighted mode	0.901 (0.708–1.147)	0.410

### Heterogeneity and pleiotropy test

The heterogeneity in IVs was evaluated by Cohran’s Q test. Cohran’s Q statistic suggested that IVs have no heterogeneity (*p* > 0.05, Table 2).

**TABLE 2:** Heterogeneity testing of IVs for the serum 25-hydroxyvitamin D level.

Method	Q	df	*p*-value
MR-Egger	81.979	100	0.9053
Inverse variance weighted	84.608	101	0.8900

**FIGURE 4:**
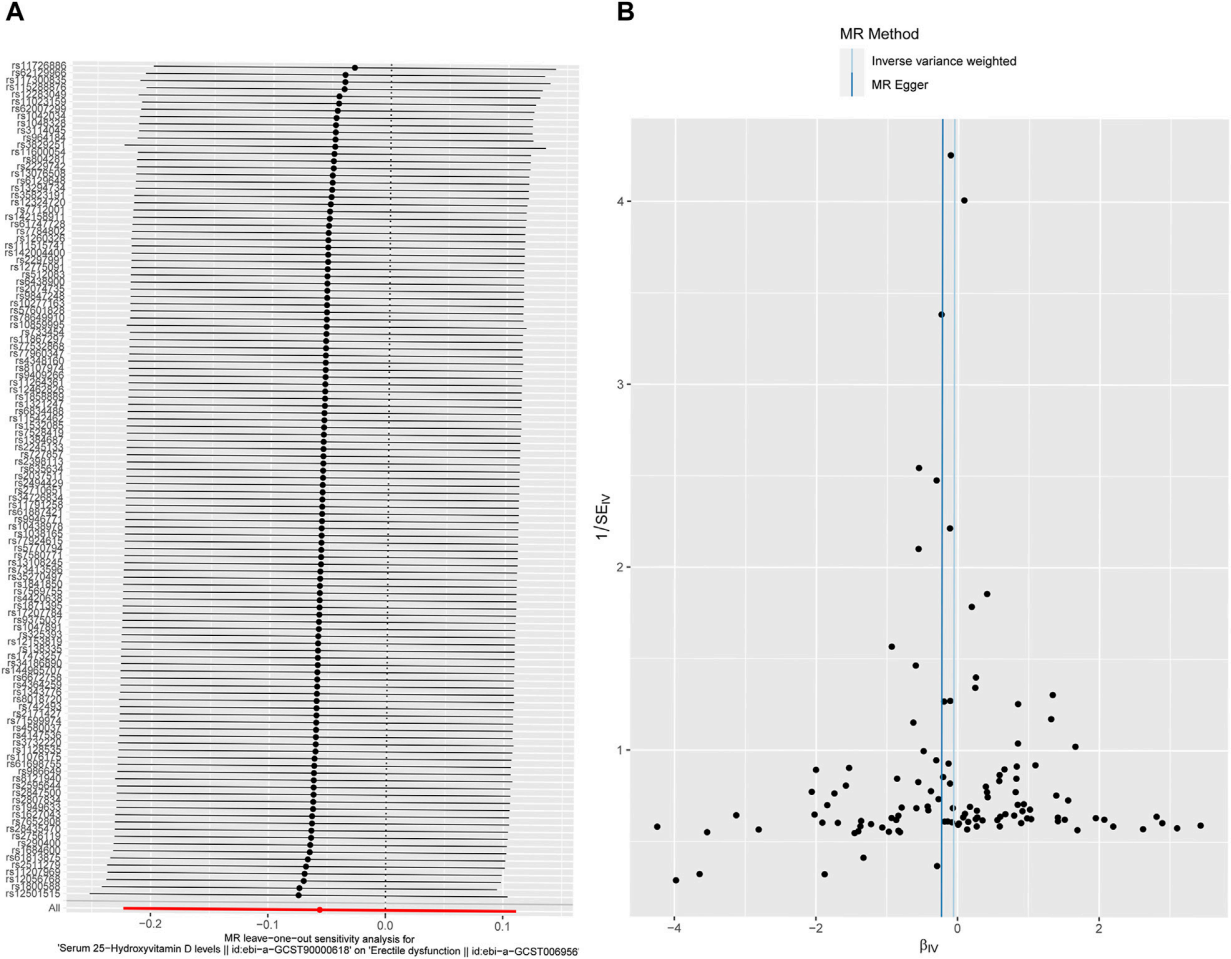
Sensitivity analysis. **(A)** Forest plot for leave-one-out analysis results. The bottom red line represents a positive IVW result. Each line represents the result of IVW when a certain SNP is removed. The bottom red line represents the result when all SNPs are included. **(B)** Funnel plot for the instrumental variables. The points represent SNPs, and the uniform and symmetrical distribution of the SNPs indicates a small heterogeneity.

MR-Egger regression was used to detect the pleiotropy of IVs. The MR-Egger regression indicated that IVs have no pleiotropy (intercept: 0.0063, se: 0.0039, *p* = 0.1081 (>0.05)). The funnel plot is similar to the funnel plot in the meta-analysis, which mainly focuses on whether the points on the left and right sides of the IVW line are roughly symmetric. If there are particular points indicating outliers, MR analysis should be performed again after removing the outlier SNPs. As shown in the figure, no outliers exist, and the funnel plot was roughly symmetrical (Figure 4B).

Therefore, the IVs we used in our study are unlikely to be associated with confounders.

### Leave-one-out analysis

The leave-one-out analysis was performed by excluding SNPs one by one and then assessing whether the results changed. The results confirmed that a particular SNP did not excessively influence the MR results (Figure 4A), manifesting that no IVs biased the causal estimates of MR.

## Discussion

Using summary level data for ED and serum 25-hydroxyvitamin D level from a large population of European descent, our study provides evidence against a causal association between serum 25-hydroxyvitamin D level and ED risk. To the best of our knowledge, this is the first MR study to explore the causal association between serum 25-hydroxyvitamin D level and ED risk.

A series of observational studies reported a strong relationship between the serum 25-hydroxyvitamin D level and ED. Barassi and colleagues found that a significant proportion of ED patients have a vitamin D deficiency and that this condition is more frequent in patients with arteriogenic etiology. They concluded that low vitamin D levels might increase the ED risk (Barassi et al., 2014). A study by Kim also showed that low vitamin D status was closely correlated with moderate to severe ED in older men with moderate to severe lower urinary tract symptoms (LUTS) (Kim and Cho, 2022). Another observational study consisting of 92 type-2 diabetes patients found that vitamin D deficiency (defined as 25-hydroxyvitamin D level of <10 ng/mL) was the independent risk factor of ED (Caretta et al., 2016). A meta-analysis study by Crafa suggested that the international index of erectile function (IIEF) score for ED was significantly worse in patients with vitamin D deficiency than in controls (Crafa et al., 2020).

These associations from observational studies and meta-analysis studies stand in contrast to our MR study findings on serum 25-hydroxyvitamin D levels in ED risk. Reverse causation and residual confounding should be considered as alternative explanations if MR studies do not support findings from observational studies (Konig and Greco, 2018). Although some confounders in observational studies were adjusted, unmeasured risk factors cannot be completely ruled out. In fact, vitamin D levels are influenced by many factors, such as intake and sun exposure (latitude, climate, personal or cultural habits of sunbathing, clothing) (Wei et al., 2019). Additionally, the serum vitamin D concentrations vary with season in subjects of all ages, being lowest during winter and early spring and highest in summer (Niculescu et al., 2017). Moreover, the methods used for vitamin D concentration measurement may also cause bias in observational studies (Dirks et al., 2023). However, most of the aforementioned studies failed to provide the related data. Furthermore, the multifactorial ED etiology was collected in a population already predisposed to ED, so pinpointing VD as an exclusive ED cause is somewhat improbable (Sudarević et al., 2017). Hence, serum vitamin D levels could be linked to various kinds of confounders, which hinders a complete adjustment in the statistical analyses in observational studies. MR utilized SNPs as instruments to estimate the causal effect between exposure and outcome, as genetic variants are randomly allocated among individuals, and they are not affected by environmental or lifestyle factors and also do not change over a lifetime. Researchers have identifiednumerous genetic variants (SNPs) associated with 25 hydroxyvitamin D concentration or ED (Revez et al., 2020; Bovijn et al., 2019; Manousaki et al., 2020). Which makes it feasible to study whether a causal relationship exists between them. Hence, although the results conflict with observational studies, our findings based on MR would be more persuasive. Similar MR research also found no causal association between serum vitamin D status and prostate cancer (Trummer et al., 2016).

Our MR analysis findings were consistent with a meta-analysis of observational studies by Wei et al. (2019), which reported no strong association between vitamin D and the risk of ED. Similarly, Sudarević and colleagues investigated whether vitamin D serum levels were associated with the presence and severity of ED, and no relationship between 25-hydroxyvitamin D concentration and ED risk or severity was observed in renal transplant recipients (Sudarević et al., 2017). However, randomized clinical trials are still urgently needed to further illustrate the relationship between serum vitamin D levels and ED risk.

### Strengths and limitations

Our MR study has several strengths. First, data from a large genetic consortium for serum 25-hydroxyvitamin D levels (n = 33,996) and ED risk (22,233 cases and 64,762 controls) enabled us to test our study hypothesis more precisely than using individual-level data from studies based on small sample size. Second, MR analysis uses genetic variation as an instrument for exposure, which can reverse the inherent causality, minimize residual confounding in observational studies, and make the causal inference more robust (Chen et al., 2023).

Our study also has some limitations. Subjects involved in this MR study were limited to European ancestry, so our findings cannot be extended to individuals of other ancestries. In addition, ED, as a highly prevalent heterogenic sexual disorder, has different subtypes according to etiology (vascular and non‐vascular ED) and severity, which were not considered in this study. Therefore, serum 25-hydroxyvitamin D levels may still have a causal relationship with certain subtypes of ED. Hence, we need to interpret the negative result of this study carefully. Further studies are necessary to explain the deeper link between VD level and ED risk.

## Conclusion

Our MR study did not support a causal relationship between genetically predicted serum 25-hydroxyvitamin D levels and ED risk in individuals of European descent.

## Data Availability

The original contributions presented in the study are included in the article/Supplementary Material; further inquiries can be directed to the corresponding authors.

## Supplementary material

SUPPLEMENTARY TABLE S1 SNPs related with confounders SNPs were examined on PhenoScanner (www.phenoscanner.medschl.cam.ac.uk) to avoid SNPs associated with potential confounders, including prostate cancer, diabetes, depression, and bipolar disorder. Finally, 14 SNPs associated with possible confounders of ED, such as prostate cancer (1 SNPs), diabetes(11 SNPs), depression and bipolar disorder(2 SNPs) were excluded in the following MR analysis. The following table shows the detail information of the 14 SNPs.

SUPPLEMENTARY TABLE S2 SNPs used in the mendelian randomization study Table S2 Detailed information of 103 SNPs employed as instrumental varibles. SNPs with a strong association with Serum 25-Hydroxyvitamin D levels were filtered based on the following criteria: genome-wide significance *P* < 5 × 10^−8^ and linkage disequilibrium r^2^ < 0.001 within a 10,000 kb window. In addition, to avoid bias from weak IVs, F-statistics of SNPs were calculated using the following formula: F-statistics = (Beta/Se)2. The values of F-statistics represented the strength of IVs.
