# Supplementary material for: Serum 25-hydroxyvitamin D level and erectile dysfunction: a causal relationship? Findings from a two-sample Mendelian randomization study
Source: Front Mol Biosci. 2024 Jun 12;11:1390814. doi: 10.3389/fmolb.2024.1390814 (PMC11200040; doi:10.3389/fmolb.2024.1390814)
Supplement: Supplementary file 1 [file Table1.docx]

**Table S1 SNPs related with possible confounders of erectile dysfunction**

SNPs were examined on PhenoScanner (www.phenoscanner.medschl.cam.ac.uk) to avoid SNPs associated with potential confounders, including prostate cancer, diabetes, depression, and bipolar disorder. Finally, 14 SNPs associated with possible confounders of ED, such as prostate cancer (1 SNPs), diabetes(11 SNPs), depression and bipolar disorder(2 SNPs) were excluded in the following MR analysis. The following table shows the detail information of the 14 SNPs.

**Table S2 Detailed information for SNPs used in this mendelian randomization study**

Detailed information of 103 SNPs employed as instrumental varibles. SNPs with a strong association with Serum 25-Hydroxyvitamin D levels were filtered based on the following criteria: genome-wide significance *P* < 5 × 10^-8^ and linkage disequilibrium *r^2^* < 0.001 within a 10,000 kb window. In addition, to avoid bias from weak IVs, F-statistics of SNPs were calculated using the following formula: F-statistics = (Beta/Se)2. The values of F-statistics represented the strength of IVs.
